# Selective Targeting and Eradication of Various Human Non-Small Cell Lung Cancer Cell Lines Using Self-Assembled Aptamer-Decorated Nanoparticles

**DOI:** 10.3390/pharmaceutics14081650

**Published:** 2022-08-08

**Authors:** Daniel Barak, Shira Engelberg, Yehuda G. Assaraf, Yoav D. Livney

**Affiliations:** 1 Lab of Biopolymers for Food & Health, Department of Biotechnology & Food Engineering, Technion, Israel Institute of Technology, Haifa 3200003, Israel; 2 The Fred Wyszkowski Cancer Research Lab, Department of Biology, Technion-Israel Institute of Technology, Haifa 3200003, Israel

**Keywords:** lung cancer, anticancer drugs, paclitaxel, targeted delivery, aptamers, nanoparticles

## Abstract

The leading cause of cancer mortality remains lung cancer (LC), of which non-small cell lung cancer (NSCLC) is the predominant type. Chemotherapy achieves only low response rates while inflicting serious untoward toxicity. Herein, we studied the binding and internalization of S15-aptamer (S15-APT)-decorated polyethylene glycol-polycaprolactone (PEG-PCL) nanoparticles (NPs) by various human NSCLC cell lines. All the NSCLC cell lines were targeted by S15-APT-decorated NPs. Confocal microscopy revealed variable levels of NP binding and uptake amongst these NSCLC cell lines, decreasing in the following order: Adenocarcinoma (AC) A549 cells > H2228 (AC) > H1299 (large cell carcinoma) > H522 (AC) > H1975 (AC). Flow cytometry analysis showed a consistent variation between these NSCLC cell lines in the internalization of S15-APT-decorated quantum dots. We obtained a temperature-dependent NP uptake, characteristic of active internalization. Furthermore, cytotoxicity assays with APT-NPs entrapping paclitaxel, revealed that A549 cells had the lowest IC_50_ value of 0.03 µM PTX (determined previously), whereas H2228, H1299, H522 and H1975 exhibited higher IC_50_ values of 0.38 µM, 0.92 µM, 2.31 µM and 2.59 µM, respectively (determined herein). Cytotoxicity was correlated with the binding and internalization of APT-NPs in the various NSCLC cells, suggesting variable expression of the putative S15 target receptor. These findings support the development of APT-targeted NPs in precision nanomedicine for individual NSCLC patient treatment.

## 1. Introduction

Lung cancer (LC) is the most common form of cancer worldwide amounting 12.3% of all cancers. The main cause of LC is tobacco smoking, with 80–90% of cases arising in cigarette smokers [1]. Over half of LC patients die within one year of diagnosis, and the 5-year survival rate is ~17.8%, based on the currently available treatments [2]. The LC death toll in 2020 attained nearly 1.8 million people and is currently the leading cause of cancer mortality worldwide [3,4]. In general, there are four major histological types which comprise the majority of LC; they are roughly split into small cell LC, and non-small cell lung cancer (NSCLC), the latter of which accounts for ~85% of all LC cases [5]. NSCLC can be further divided into several histological subtypes: adenocarcinoma (AC), squamous cell carcinoma, adenosquamous cell carcinoma, large cell carcinoma, and sarcomatoid carcinoma, with AC accounting over 40% of NSCLC cases [6,7]. Current treatment options include surgery, immunotherapy, chemotherapy, radiotherapy, and adjuvant therapy [2]. Non-targeted chemotherapy is administered in combination with surgery in most patients, albeit inflicting serious side effects related to the cytotoxicity of anticancer drugs to rapidly dividing cells (e.g., bone marrow, epithelial cells of the gastrointestinal tract, hair follicles, and sperm cells). These deleterious effects damage the host’s defense mechanisms, which are often already compromised by the underlying cancer. Cancer patients are therefore highly susceptible to infections, especially bacterial viral and fungal infections. Furthermore, all types of infections are associated with higher rates of morbidity and mortality in cancer patients receiving non-specific chemotherapy treatment [8].

Despite its many side effects, paclitaxel (PTX) is currently one of the most effective chemotherapeutic drugs used for the treatment of various cancers including breast, ovarian, lung, head and neck cancer and NSCLC [9]. PTX promotes intracellular polymerization of tubulin, thereby causing cell death by disrupting the normal microtubule dynamics required for cell division and vital interphase processes [10,11,12]. Nonetheless, administration of PTX has been associated with multiple toxicities [13]. Formulation aids such as Cremophor EL are currently used in the clinic for various poorly water soluble chemotherapeutic drugs such as PTX, since the hydrophobic nature of many antitumor agents limits their administration at high doses [14]. Cremophor EL is not an inert agent by itself, exerting a range of adverse biological effects, some of which have serious clinical implications [15]. Common short- and long-term side effects of these treatments include severe anaphylactoid hypersensitivity reactions, hyperlipidemia, abnormal lipoprotein patterns, aggregation of erythrocytes and peripheral neuropathy [16].

The untoward toxicity of PTX, its high lipophilicity and the toxic side effects, call for the development of novel, selectively targeted, and non-immunogenic modalities to eliminate cancer cells in patients, using a minimally invasive approach and without harming normal cells. For example, micellar nanoparticles made of amphiphilic polymers can be used for drug entrapment and solubilization, and can be targeted by specific tumor-targeting ligands [17].

Recent nanomedicine studies have shown it to be an avenue of incredible potential for the development of specific tumor-targeted drug delivery systems [18,19,20,21]. This continuously developing therapeutic approach is expected to revolutionize cancer treatment and management [14]. More precisely, nanoscale drug delivery systems hold great promise in successfully formulating and enhancing the therapeutic efficacy of a large number of anticancer agents [14]. Nanoparticles (NPs) are known to enhance biodistribution, increase therapeutic efficacy and reduce untoward toxicity of potent anticancer drugs. Their superior biocompatibility, ability to protect encapsulated compounds from metabolism and degradation, ability to deliver therapeutics to cancer cells in vivo, and/or to passively target tumors via the enhanced permeability and retention (EPR) effect [22,23,24], render NPs highly promising delivery vehicles [14]. Currently, several types of NP-based drug delivery systems are being developed to address the problems raised above and to allow for a more facile, precise, and efficacious LC treatment. For example, several types of nanocarriers that are currently studied for drug and gene delivery applications include liposomes, micellar NPs, nanostructured lipid carriers, polymeric NPs and more [20,21,25,26]. Recently, NPs made of polymers such as polyethylene glycol (PEG), poly (lactic acid) (PLA), poly (lactic-co-glycolic) acid (PLGA), gelatin, albumin, chitosan, polycaprolactone (PCL), poly-alkyl-cyanoacrylates, and their block copolymers have gained popularity in use, because of their controlled and sustained release properties, subcellular size, and biocompatibility [27]. Polymeric NPs have also been shown to enhance the chemo- and radio-therapeutic efficacy of anticancer agents [28]. The amphiphilic nature of some of the polymers constructing the NPs, particularly block copolymers, allows them to self-assemble into micelles and thus entrap and carry hydrophobic drugs such as PTX through the blood circulation [29,30].

To allow for a more efficacious drug delivery and pharmacological activity against the tumor cells, specific active targeting ligands are used by decorating the NPs. These targeting ligands include, e.g., vitamin E derivatives, biotinylated epidermal growth factors, folic acid, antibodies, and aptamers. They allow obtaining high targeting precision and selectivity for the tumor cells, thereby markedly reducing the side effects of the chemotherapeutic drug cargo [31].

Aptamers (APTs), which are usually single-stranded DNA or RNA molecules, are emerging as promising targeting ligands for both cancer diagnostics and therapy [32]. APTs are comparable to antibodies regarding specificity and affinity for their target, which is typically a cell-surface protein or receptor [33]. APTs are much smaller than antibodies and are comparable in their molecular weight to peptides (1–5 kDa). APTs can be chemically produced for any cell-surface target, e.g., using a methodology called “SELEX” (Systematic Evolution of Ligands by EXponential Enrichment) [34]. APTs possess several desirable features over other targeting strategies, mainly their small size, low immunogenicity, and high specificity [35]. APTs can be coupled to diagnostic or therapeutic agents or to polymers, such as PEG, to enable selective targeting [33]. However, aptamers, as oligonucleotides, are susceptible to nuclease degradation, which may affect in vivo function. Thus, aptamer modifications are being added to thwart and protect them from nuclease degradation, leading to an improvement in their stability in biological environments [36,37].

We have recently demonstrated the selective targeting and eradication of human A549 NSCLC cells with PEG-PCL (poly (ethylene glycol)-poly (ε-caprolactone)) block co-polymer NPs, without harming healthy cells [38]. These NPs were conjugated to the APT S15, an 85-base-long single-stranded DNA, as the targeting ligand. To create NPs suitable for endocytosis by target NSCLC cells, we aimed at forming NPs with a size < 50 nm. At 70 μM PEG-PCL, NPs formed with a mean size of 30 ± 1 nm. The drug loading capacity and encapsulation efficiency of PTX with increasing drug: polymer molar ratios were recently determined [38]. The APT-NPs morphology was studied with cryogenic transmission electron microscopy (Cryo-TEM), NPs entrapping PTX were observed, with a clear core and shell structure. The in vitro release of PTX from APT-NPs was assessed [38]. APT-NPs loaded with PTX were shown to have selective cytotoxicity to target A549 cells, entering the cells via clathrin-mediated endocytosis [38,39].

In the current study, we aimed at characterizing the S15 APT-NP delivery system, in terms of selectivity and cytotoxicity to various model human NSCLC cell lines. After reviewing the relevant literature, to the best of our knowledge, no previous study had investigated the sensitivity of various NSCLC subtypes to APT-decorated NPs. Our findings can serve as the initial basis for the identification of the putative surface receptor that specifically binds and internalizes the S15-decorated NPs in NSCLC cells. Further studies can also pave the way toward tailoring NPs for a personalized medicine treatment of individual cancer patients.

## 2. Materials and Methods

### 2.1. Materials

The tumor cell lines used in the current study were generously provided by the following labs: AC H2228 [40] NSCLC cell line was provided by Prof. Arie Admon (Faculty of Biology, Technion, Haifa, Israel), lung carcinoma H1299 [41] and AC H1975 [42] cell lines were provided by Dr. Iris Kamer (Shiba Medical Center, Ramat-Gan, Israel) and the AC H522 [43] cell line was provided by Prof. Yosef Yarden (Dept. of Biological regulation, Weizmann Institute of Science, Rehovot, Israel).

All chemicals and cell culture reagents were obtained from Sigma-Aldrich (Merck) unless otherwise stated. PEG-PCL (5:2.5) KDa block-copolymer containing an addition of a carboxylic group at the PEG terminus, was custom synthesized (Creative PEGWorks, Durham, NC. Item number: DCD-5k2500, lot number: ZXM07050). Qdot™ 545 ITK™ Carboxyl QDs were purchased from Thermo Fisher Scientific (Waltham, MA, USA), lot number: 2273708. The S15 aptamer with the following nucleotide sequence: 5′-NH2-ACG CTC GGA TGC CAC TAC AGG CTA TCT TAT GGA AAT TTC GTG TAG GGT TTG GTG TGG CGG GGC TAC TCA TGG ACG TGC TGG TGA CidT-3′ was purchased from BioSpring Gesellschaft für Biotechnologie mbH, (Frankfurt, Germany), lot number: 257225_E. idT = inverted deoxythymidine incorporated at the 3′-end leading to a 3′-3′ linkage for protection against exonuclease degradation, see Supplementary Information [39].

### 2.2. Cell Cultures

Human NSCLC cell lines: A549, H2228, H1299, H1975, and H522 were maintained in RPMI-1640 medium, supplemented with 10% fetal bovine serum (FBS), 2 mM glutamine, 100 µg/mL penicillin and streptomycin (Biological Industries, Beit Ha-Emek, Israel). Cells were incubated under a controlled temperature of 37 °C in a humidified atmosphere of 5% CO_2_, and their monolayer status was followed using standard light microscopy. Freezing and thawing of cells was carried out using a previously published protocol [44].

### 2.3. Methods

#### 2.3.1. Preparation of Cy7-Labeled APT-NPs

**Figure 1:**
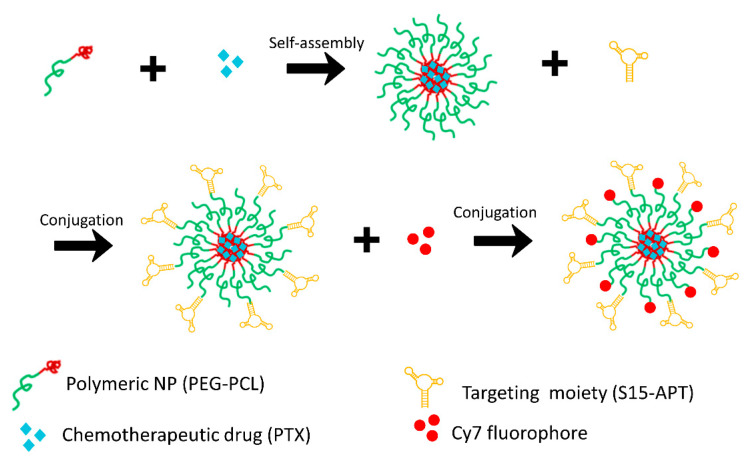
Schematic structure of the Cy7-labeled APT-NPs and their preparation steps.

Self-assembled PEG-PCL micelles were prepared by surfactant-free nanoprecipitation [45,46] with minor modifications of a previous protocol [38]. PEG-PCL was dissolved in acetonitrile (ACN), then added dropwise into a 0.8 mL–0.9% saline solution (Biological Industries, Kibbutz Beit-HaEmek, Israel) to a final concentration of 70 µM, and stirred at 161× *g* until complete evaporation of ACN was achieved. Thereafter, 1.3 µM EDC (1-Ethyl-3-(3-dimethylaminopropyl) carbodiimide) and 0.2 µM NHS (N-Hydroxy succinimide) freshly dissolved in saline, were added to the mixture for 10 min to create the NHS ester group at the ends of the PEG chains, in the well-established carbodiimide conjugation reaction [47,48]. Then, 0.03 µM of amine-modified S15-APTs, which were previously filtered through a 4 µm syringe filter, were added and left under stirring for 2 h to create the amide linkage with the PEG-NHS tail. The APT-conjugated polymeric NPs were ultra-filtered twice (1700× *g*, 4 min, 4 °C) through a 100 kDa MWCO membrane (Merck, Darmstadt, Germany) to remove unbound free aptamers. The carbodiimide EDC-NHS conjugation reaction was repeated using 0.5 µM Cy7 (Cyanine7)-amine fluorescent dye, to attach it to the carboxylic end of yet undecorated PEG blocks, and was followed by a similar filtration step. NPs were stored in the dark at 4 °C until the incubation with cells. A schematic structure of the Cy7-labeled APT-NPs and their preparation steps are depicted in Figure 1.

#### 2.3.2. Particle Size Distribution Analysis and Cryo-TEM Imaging

Particle size distribution of the APT-NPs was determined by dynamic light scattering (DLS), using a DLS analyzer (NICOMP^TM^ 380, Particle Sizing System (PSS), Inc., Santa Barbara, CA, USA), as previously described [38]. APT-decorated NPs were observed by Cryo-TEM imaging using FEI Talos 200 C electron microscope (accelerating voltage 200 kV) at a low dose imaging mode. Specimen preparation was conducted as previously described [49]. Images were acquired by a FEI Falcon III direct-imaging camera, using “phase-plates” (FEI), to enhance image contrast [50].

#### 2.3.3. Characterization and Comparison of the Specificity of APT-NPs to Various NSCLC Cell Lines Using Confocal Laser Microscopy

Specific internalization of APT-NPs by different NSCLC cell lines including H2228, H1299, H1975 and H2228 was determined using confocal laser microscopy imaging. 120 µL of each cell suspension were seeded on μ-slides VI 0.1 (Ibidi, Martinsried, Germany) at 50% confluency (15 × 10^4^ cells/mL) and incubated overnight to allow for cell attachment. Cells were then washed with phosphate-buffered saline (PBS) pH 7.4. For internalization studies, APT-NPs were diluted 1:5 (*v*/*v*) in FBS-free medium and incubated with the cells for 2 h at 37 °C. Cells were washed three times with PBS to remove unbound APT-NPs. Cells were then incubated with 10 µg/mL Hoechst 33342 in growth medium for 20 min to achieve nuclear DNA staining. Fluorescence was studied using an inverted laser scanning confocal microscope (Zeiss LSM 710). Two fluorescence channels were used during all image capturing: 405 nm excitation laser for the blue viable DNA dye Hoechst 33342 (461 nm max emission) and 639 nm excitation laser for the red Cy7 dye (767 nm max emission). Confocal microscopy and data acquisition were performed using a ×40 magnification water-immersion lens. Further analysis and image processing was performed using the ZEN 2011 microscope imaging software by ZEISS.

Selective binding of S15 APTs to the studied NSCLC cell lines was performed similarly to the internalization procedure described above with minor modifications. Cells were exposed to 10 µg/mL Hoechst 33342 in growth medium for 20 min before incubation with NPs to allow for nuclear staining. Following NPs dilution in FBS-free medium, they were added to the cells and kept at 4 °C for 50 min. The cells were then washed three times with PBS and kept on ice until fluorescence microscopy analysis was performed.

#### 2.3.4. Image Analysis

To obtain quantitative measurements from the captured photos, IMARIS 9.8 software for analysis of image data was used (Oxford instruments, Oxon, UK). The red fluorescence channel detection threshold was set in the range of 10–100 relative fluorescence units for all presented images, which allowed us to acquire the values of intracellular mean fluorescence intensity (MFI) and the mean value for the number of fluorescent endolysosomes per cell (FEPC).

#### 2.3.5. Characterization of Active S15 APT-NPs Internalization by NSCLC Cell Lines

##### Conjugation of QDs

S15 aptamers were conjugated to carboxy terminals of QDs, as previously described [51]. Qdot^®^ 545 ITK™ carboxyl QDs, at a final concentration of 1 µM, were added to 0.75 nmol S15 filtered APTs in a final volume of 100 μL of freshly prepared 10 mM sodium borate buffer pH 7.4—and kept in the dark. Following 5 min incubation at room temperature, 3 μL of a 10 mg/mL EDC solution (freshly prepared in 10 mM sodium borate buffer at pH 7.4) were added to the mixture and kept for 18 h at room temperature with continuous stirring at 40× *g*. After the reaction took place, the vial was centrifuged and the APT-QDs solution was ultra-filtered (1700× *g*, 4 min, at 4 °C) via centrifugal concentrators (100 kDa MWCO PES, Vivaspin, Sartorius Stedim Biotech GmbH, Göttingen, Germany) to remove free unconjugated aptamers. The purification step was repeated 4 times using 50 mM sodium borate buffer at pH 8.3 to increase pH and maintain the APT-QDs concentration.

##### Flow Cytometric Analysis

H2228, H1299, H1975 and H522 cells were plated in 24-well plates at approximately 50% confluence, depending on their respective growth rates. Following 18 h of incubation at 37 °C, cells were first washed with 1 mL PBS to remove serum remnants and dead cells. Next, the previously prepared APT-QD’s solution was diluted 1:4 (*v*/*v*) in FBS-free medium and incubated with the cells for 1 h at 37 °C or 4 °C. An unstained control group, which was not exposed to the APT-QDs, was maintained simultaneously at 37 °C for each of the tumor cell lines. Following incubation, cells were washed 3 times with 1 mL PBS and incubated with 0.3 mL HBSS buffer containing 0.05% trypsin and 0.53 mM EDTA for 15 min. After incubation, 0.3 mL of FBS-containing protease inhibitors, including α1-antitrypsin (Biological Industries, Beit-HaEmek, Israel) [52], were added, and the cells were then transferred to 2 mL Eppendorf microfuge tubes and centrifuged at 800× *g* for 3 min, at room temperature. The supernatant was aspirated, and the sedimented cells were then resuspended in 0.5 mL growth medium and transferred into flow cytometry tubes through a 35 μm strainer cap. Flow cytometry analysis was performed using the BD LSR II flow cytometer (BD Biosciences). The QDs were excited at 355 nm, while their emission was detected using a 530/30 nm filter (green). Raw data processing and further downstream analysis was carried out using the flow cytometry analysis tool FCS Express™ 7.12.0005.

#### 2.3.6. Cytotoxicity Assays

##### Preparation of Aptamer-Decorated Nanoparticles Harboring a Cytotoxic Drug

S15 APT polymeric NPs were prepared similarly to the above protocol, with minor modifications, to allow for the efficient entrapment of the hydrophobic drug PTX in the core of the NPs. To this end, final concentrations of 70 µM PEG-PCL and 14 µM PTX were dissolved simultaneously in ACN. The ACN solution was then added dropwise into 1 mL of saline and stirred at 161× *g* until complete ACN evaporation was achieved. Exonuclease protected S15 APTs (modified with a 3′ deoxy thymidine cap [53]) were conjugated to the PEG carboxy terminal as described above, using EDC and NHS freshly dissolved in saline. The conjugated NPs were ultra-filtered and repeatedly concentrated (2200× *g*, 4 min, 4 °C) until a concentration of 60 µM was achieved.

##### Cytotoxicity Assay

The selective cytotoxicity of PTX entrapped within ATP-NPs was studied in the various tumor cell lines. First, a confluency calibration was performed: cell lines were seeded and grown on a 96-well plate at different cell densities for 72 h, to calibrate the minimal necessary cell density for full confluency at the exposure time. Seeding cell densities for each tumor cell line were determined to be 4 × 10^4^, 1.7 × 10^4^, 1.8 × 10^4^, and 2.5 × 10^4^ cells/mL for H2228, H1299, H1975 and H522, respectively (cell counting was performed using a hemocytometer). Cell lines were seeded in their appropriate calibrated density in 96-well plates- 100 µL of cell suspension in each well. After an incubation period of 24 h, with the same nanoparticle concentration in all cell lines, the growth medium was replaced by FBS-free medium and the APT-NPs entrapping PTX were serially diluted in the wells to obtain 11 increasing concentrations of 0.3–30 µM. Cells in the 12th column were left drug free and were used as the 100% cell viability control reference. The procedure was performed in 9 replicates for each of the cell lines and the results presented were obtained from five independent experiments. Cell exposure to the drug-loaded NPs was for 2 h at 37 °C, followed by three washes with growth medium to remove the remaining non-internalized APT-NPs/PTX. The cells were then incubated for additional 72 h in growth medium, to allow for PTX to elicit its cytotoxic effect. Following this incubation period, cell viability was assessed using a colorimetric XTT-based cell proliferation assay [54] (Biological Industries, Beit-HaEmek, Israel). The XTT reagent (2,3-Bis-(2-Methoxy-4-Nitro-5-Sulfophenyl)-2H-Tetrazolium-5-Carboxanilide) and activation phenazine methosulfate reagent (N-methyl dibenzopyrazine methyl sulfate) were thoroughly thawed. The reagent was mixed with the intended activator at a 1:50 (*v*:*v*) ratio. Then, 33 µL of the mixture were added to 67 µL of growth medium in each well, and the cells were incubated at 37 °C for 20 min. Blank samples were performed in triplicates by adding the same volumes specified above to cell-free wells. Following 20 min of incubation, gentle orbital shaking was applied to the plates to evenly distribute the orange color of the obtained formazan in each well. Thereafter, the absorbance of the formazan dye formed by the metabolically active cells was measured at 450 nm, using a scanning multiwell spectrophotometer (Biotek Microplate Reader, Winooski, VT, USA). Data collection and its initial handling was performed using the BioTek Gen5 v3.11 software. The collected data was further analyzed using a nonlinear curve fitting model (Hill1) [55] with the OriginPro 9.0 graphing and analysis software for dose–response curve represented according to Equation (1) [56]:

P = P_∞_ + (P_0_ − P_∞_)∙([D]^n^/(IC_50_)^n^ + [D]^n^) (1)

In Equation (1), P is the percentage of viable cells; P_∞_ is the minimal percent of live cells at theoretical infinite drug concentration which results in zero cell viability; P_0_ is the maximal percent of surviving viable cells in the absence of drug (when its concentration is zero, as in the control column, cell viability is 100%); [D] is a the drug concentration; IC_50_ is the drug concentration at which half-maximal inhibitory effect in cell growth is achieved; n represents the Hill slope coefficient, indicating the steepness of the dose–response curve. Curve fitting was performed at a significance level of α = 0.05.

## 3. Results and Discussion

### 3.1. Size Distribution of NPs and Cryogenic Transmission Electron Microscopy (Cryo-TEM) Analysis

**Figure 2:**
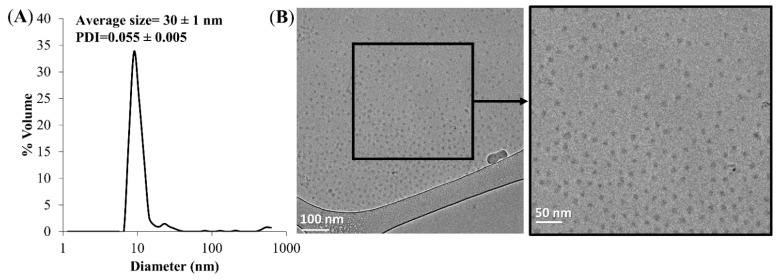
(**A**) Particle size distribution of APT functionalized PEG-PCL NPs. Average NP size = 30 ± 1 nm; PDI = 0.055 ± 0.005 (the measurements are based on duplicates). (**B**) Cryo-TEM image of APT-functionalized PEG-PCL NPs.

The average diameter of the APT-NPs was determined to be 30 ± 1 nm using DLS. The polydispersity index (PDI) was calculated using Equation (8) in reference [57] and was found to be 0.055 ± 0.005 (Figure 2A). A PDI value < 0.1 indicates a stable and monodisperse NP population [58]. The ideal size, at which NPs escape renal exclusion and RES uptake, allowing for enhanced cell permeability into solid tumors via passive diffusion, and supporting endocytosis into malignant cells is ~10–50 nm [59,60]. Cryo-TEM analysis further supported these data and revealed the morphology of APT-decorated PEG-PCL NPs encapsulating 14 µM PTX (Figure 2B); the APT-NPs appeared as small stable spheres.

### 3.2. Specificity of Binding and Internalization of APT-NPs in Various Human NSCLC Cell Lines

**Figure 3:**
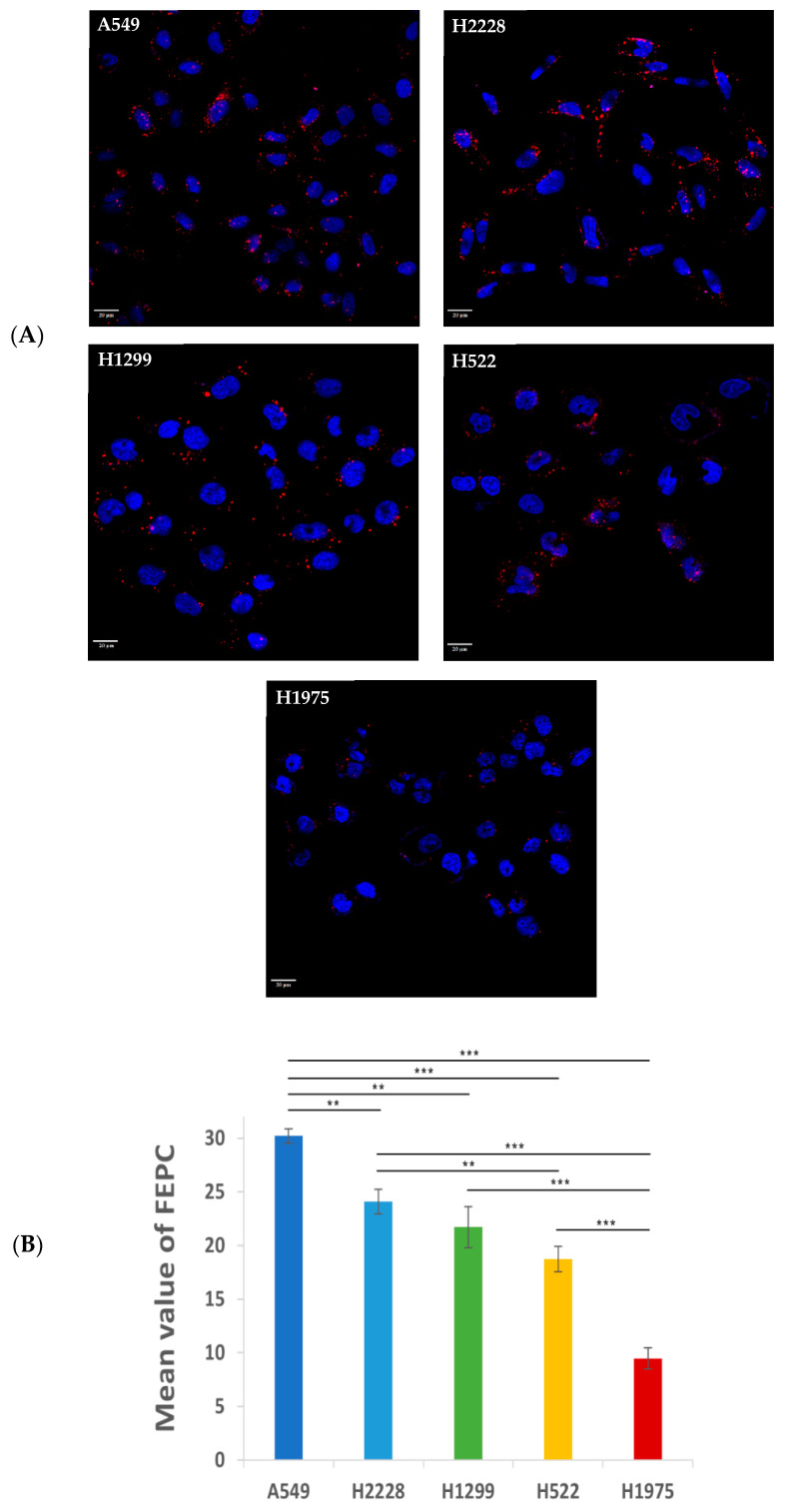
(**A**) Variable internalization of APT-NPs by various NSCLC cells at 37 °C. Confocal laser microscopy images of NSCLC subtypes exposed to PEG-PCL NPs decorated with S15 APTs. The tumor cell lines were H2228, H1299, H522 and H1975. Cells were incubated at 37 °C, for 2 h, in FBS-free medium, containing APT-NPs labeled with Cy7 (red). Nuclear DNA was labeled with Hoechst 33342 (2 μg/mL) (blue). A 20 μm scale bar is shown. (**B**) Mean FEPC values ± SE of Cy7-labeled S15 APT-NPs. Values were determined using IMARIS software in the images shown in (**A**). ** *p* < 0.01, *** *p* < 0.001, unpaired, two-tailed *t*-test.

The specificity of binding and internalization of APT-NPs in various human NSCLC cell lines was studied using confocal laser microscopy. Following an incubation with 14 µM S15 APT-NPs for 2 h at 37 °C, a variability in the red fluorescent intracellular vesicles was observed among the NSCLC cell lines H2228, H1299, H522 and H1975 (Figure 3A). This was reflected in the numbers of accumulated FEPC, containing the Cy7-labeled NPs (Figure 3B) [38,61]. H2228, H1299, H522 and H1975 cells displayed FEPC values of 24.1, 21.7, 18.7 and 9.5, respectively, whereas human NSCLC A549 cells, which were previously found to be well targeted by these NPs [38], were found to have a remarkable mean FEPC value of 30.2. The observed difference in the internalization capacity of the APT-NPs by these NSCLC cells, suggests a variable surface expression of a putative receptor recognized by the S15 APT-NPs, which accordingly impacts their uptake abilities. Indeed, physiological differences between subtypes of NSCLC cell lines have been previously studied and characterized. Molina-Romero Camil et al., found differential gene expression patterns amongst several subtypes of AC [62]. They were able to identify a gene expression signature consisting of 13 genes which affected different functional networks related to six distinct biological categories: lipid metabolism; biochemistry of small molecules; vitamin and mineral metabolism; DNA replication; recombination and repair; and cell cycle. Additional studies have concluded that different molecular subtypes of NSCLC exhibit diverse gene mutation rates in key genes, including EGFR, KRAS, STK11, and TP53, chromosomal instability, regional copy number, and genome-wide DNA methylation [63]. In addition, lung cancer subtypes, patient behavior such as smoking, and/or patient germline sequence may further modulate gene expression patterns. The intrinsic properties of cancer cell types may promote specific genomic alterations due to a hyper-mutator phenotype, along with the selective advantage that a specific mutation confers upon a specific cell type [63]. Despite the expected molecular subtype variance, even H522 and H1975 cells, which displayed the lowest level of internalization of S15 APT-NPs, still appeared to be substantially targeted by these S15 NPs. In contrast, in similar experiments we recently conducted with the same S15 APT-NPs, we found that neither normal lung epithelial BEAS2B cells, nor cervical carcinoma HeLa cells, colorectal adenocarcinoma CaCo-2 cells, neonatal foreskin FSE cells and embryonic kidney HEK-293 cells, showed any cellular fluorescence when incubated with these S15 APT-NPs [38]. These cumulative findings suggest that S15 APT-NPs demonstrate affinity and selectivity toward various NSCLC subtypes, but at the same time, they have no affinity toward healthy tissues, which can be highly important in the clinical setting. These findings are also supported by previous studies, which demonstrated that mucin 1 and S15 APTs have the ability to target molecular abnormalities, which are specific for NSCLC adenocarcinoma, while displaying a slightly lower targeting ability for large cell carcinoma, and almost no targeting ability to other cancerous lung cells nor to other types of cancer cells [39,64]. Powell et al., reported that the A6-APT NP system for the selective delivery of P-gp-targeted siRNA (aimed at P-gp knockdown) into breast cancer cells had been evaluated using seven different cell lines. Using fluorescence microscopy, they found that different expression levels of the A6-APT target receptor Her-2 led to distinct internalization levels in the cells [65]. Therefore, these data support our findings shown in Figure 3.

Consistently, a previous theranostic study by Kim et al., reported that hydrophobic QDs were first effectively incorporated into lipid bilayers, and then therapeutic siRNAs were complexed with QD-lipid nanocarriers (QLs) [66]. Thereafter, anti-EGFR aptamer-lipid conjugates were inserted into the QLs for triple negative breast cancer (TNBC) targeting (aptamo-QLs). TNBC-targeted aptamo-QLs were compared to anti-EGFR antibody-coupled immuno-QLs. The in vitro delivery of therapeutic siRNAs and QDs to target TNBC cells was verified by flow cytometry and confocal microscopy. The in vivo tumor targeting of siRNAs and their therapeutic efficacy were evaluated in mice harboring TNBC MDA-MB-231 tumors. Both types of EGFR-targeting QLs displayed enhanced delivery to target TNBC cells, yielding more effective gene silencing and enhanced tumor imaging compared to non-targeted control QLs. Furthermore, combination therapy with Bcl-2 and PKC-ι siRNAs loaded into the anti-EGFR QLs was efficacious in inhibiting both tumor growth and metastasis.

**Figure 4:**
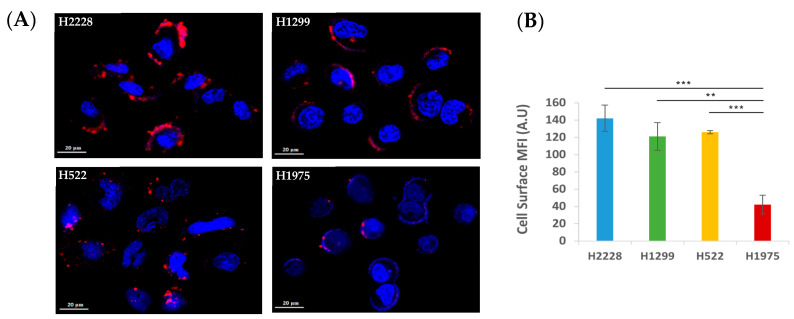
Cell surface binding of APT-NPs at 4 °C differs among NSCLC cell lines. (**A**) Confocal laser microscopy images of NSCLC cell lines incubated with PEG-PCL NPs decorated with S15 APTs. The studied tumor cell lines were H2228, H1299, H522 and H1975. Cells were incubated at 4 °C, for 50 min, in FBS-free medium, containing APT-NPs labeled with Cy7 (red). Nuclear DNA was labeled with Hoechst 33342 (2 μg/mL) (blue). (**B**) Mean fluorescence intensity (MFI) values of Cy7-labeled NPs bound to cells in panel A were determined using IMARIS software. Error bars represent SE. ** *p* < 0.01, *** *p* < 0.001, unpaired, two-tailed *t*-test.

Specific binding of the S15 APT-NPs to the different cell lines was further explored, to confirm that their uptake by the cells is an active internalization process. Cells were incubated with Cy7-labeled APT-NPs for 50 min, at 4 °C, a temperature at which surface binding is retained, whereas endocytosis is prevented. Cells were then gently washed with ice-cold PBS to remove free unbound NPs. Using confocal laser microscopy, a fluorescent layer was observed colocalized with the plasma membrane, whereas fluorescent endosomes were essentially absent from the cytoplasm (Figure 4A). H2228 displayed the highest mean fluorescence intensity (MFI) of attached NPs, followed by H522 and lastly H1299 and H1975 cells (Figure 4B), in accord with their respective uptake ability shown above (Figure 3), excluding H522 cells which displayed a higher apparent specific binding of NPs than H1299. The higher MFI observed with H522 cells might be attributed to the partial internalization of NPs, which can be seen as cytoplasmic fluorescent dots near the cell nuclei (Figure 4A). A possible explanation is that the cells were transiently exposed to a temperature slightly higher than 4 °C, while being transferred to the confocal microscope, and therefore, some internalization might have occurred.

These findings suggest that the mechanism of entry of the NPs into the cells is based upon a putative receptor expressed on the cell surface, which specifically binds S15-decorated NPs, since unbound NPs were completely washed out [67]. Similarly, previous studies have also shown the ability of aptamers to bind to specific plasma membrane cell receptors, such as the receptor tyrosine kinases ErbB2, epidermal growth factor (HER)-3, as well as CD4 [68,69,70]. Thus, the possibility of a nonspecific adsorptive uptake route, such as micropinocytosis, or diffusion through the lipid bilayer, is highly unlikely, suggesting that an active internalization process was taking place, as was similarly shown for ligand conjugated polymeric NPs, targeting a specific entity on the plasma membrane of NSCLC and prostate cancer cells [71,72]. Thus, the entry mechanism into these cells is suggested to be a clathrin-mediated endocytosis, as we have previously described for A549 cells [73,74].

### 3.3. Further Exploration of the Selective Binding and Internalization of the S15-Decorated NPs

**Figure 5:**
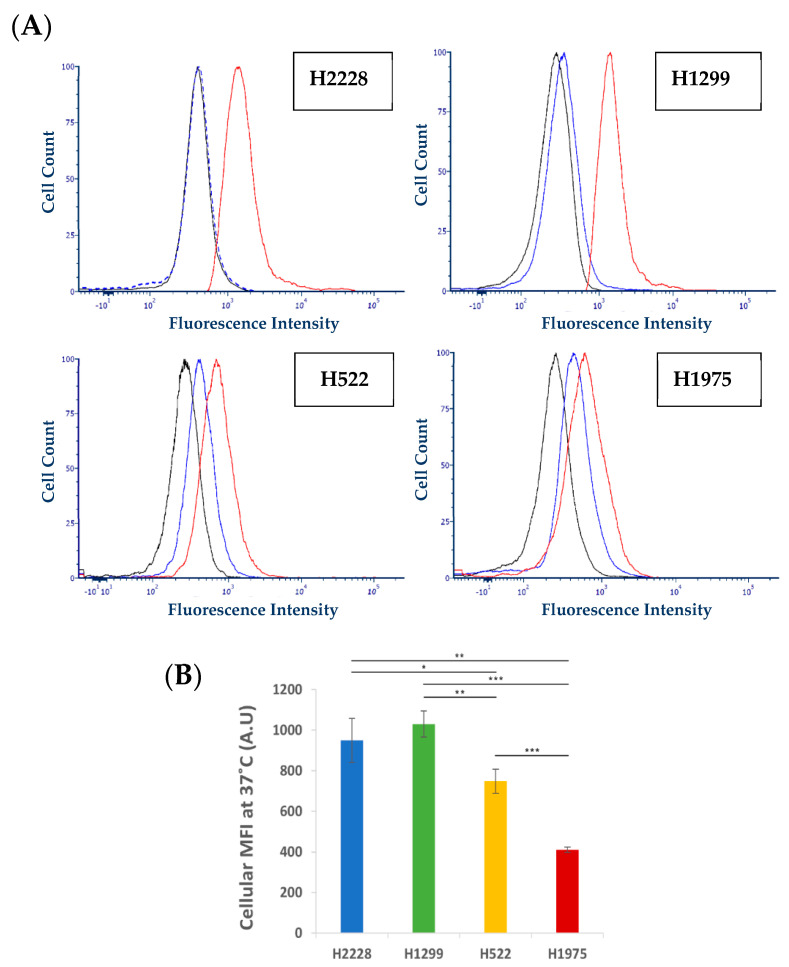
Internalization of APT-QDs in various NSCLC cell lines. (**A**) Cellular fluorescence profiles: APT-QDs exposed to the NSCLC cell lines H2228, H1299, H522 and H522 at 37 °C and 4 °C for 1 h, followed by a 15 min trypsin treatment to remove membrane bound APT-QDs. Black line: control, unstained cells. Blue line: cells incubated at 4 °C. Red line: cells incubated at 37 °C. (**B**) Mean intracellular fluorescence intensity values ± SE after APT-QD exposure at 37 °C. Autofluorescence was subtracted. * *p* < 0.05, ** *p* < 0.01, *** *p* < 0.001, unpaired, two-tailed *t*-test.

Flow cytometry analysis was performed to verify the observed differences in the binding ability and internalization of S15 NPs into NSCLC cell lines. The four NSCLC cell lines were pre-incubated at 37 °C, a temperature at which the cells were shown to actively internalize the NPs to different extents. In contrast, at 4 °C (Figure 4A), active internalization is known to be abolished [75], as we have previously shown using the same NPs with A549 NSCLC cells [51]. Following 1 h incubation with S15-APT QDs, cells were detached by trypsinization, and cellular MFI was determined using flow cytometry. We found a minor shift in cellular fluorescence intensity [76] in the profile of all examined cells incubated at 4 °C, compared to their autofluorescence level Figure 5A). This shift is also consistently much smaller compared to the large shift in the histograms from the experiments carried out at 37 °C, compared to the autofluorescence of unstained cells. These results reveal the specific binding of the S15 APTs to the cell surface of NSCLC cells, as was previously demonstrated [39]. Since trypsin is well known to remove large amounts of both glycoproteins and glycosaminoglycans from the cell surface [77], this trypsin treatment, routinely used for cultured cell detachment, most likely resulted in the removal of the putative surface receptor protein which specifically interacted with the S15-APT QDs, thus eliminating their specific binding to the target cells. These findings are also consistent with the results obtained with confocal microscopy analysis (Figure 3 and Figure 4), where the internalization of S15 NPs at 4 °C, was markedly blocked and the NPs exhibited cell surface binding, while being readily internalized at 37 °C. This supports the conclusion that the mode of entry of NPs into the cells, is a temperature-dependent process, which is characteristic of an active (ATP-dependent) endocytosis process, similarly to the results of our previous study with A549 cells, targeted by the same NPs [51]. The prominent up-shift in cellular fluorescence intensity observed at 37 °C is a result of the APT-QDs being actively internalized by the cells prior to the trypsin treatment. The net cellular fluorescence of cells incubated at 37 °C implies a difference in their active internalization ability (Figure 5B). Although the net cellular fluorescence values do not significantly differ from each other, they demonstrate a similar trend of internalization ability to what was observed in Figure 3A. H1975 cells showed the lowest net cellular fluorescence intensity which was 2.5-fold lower than that of H1299 cells, which exhibited the highest fluorescence. The same general correlation of MFI and FEPC values was observed for the four tumor cell lines, both in the confocal laser microscopy (Figure 3 and Figure 4) and flow cytometry analyses (Figure 5), further strengthening our observations [39,51]. Consistently, cell-type specific binding (at 4 °C) and internalization (at 37 °C) was previously observed by Ferreira et al., for three types of APTs directed against MUC1. Using flow cytometry, cell surface binding revealed different binding affinities to the distinct target cells (MCF-7, T47D, PANC-1) and no binding to non-target Chinese hamster ovary cells. The internalization of APTs at 37 °C was determined using confocal laser microscopy and is consistent with our results as different fluorescence intensities could be observed for the four cell lines we studied [78].

### 3.4. Cytotoxicity of APT-NPs to NSCLC Cell Lines

Prior to investigating cytotoxicity of drug-loaded APT-NPs, we first verified that the drug-free APT-NP are devoid of cytotoxic activity. For this purpose, we chose A549 cells, which, according to Figure 3, displayed the highest uptake of the APT-NPs, and hence, they are expected to be most sensitive to these APT-NPs.

**Figure 6:**
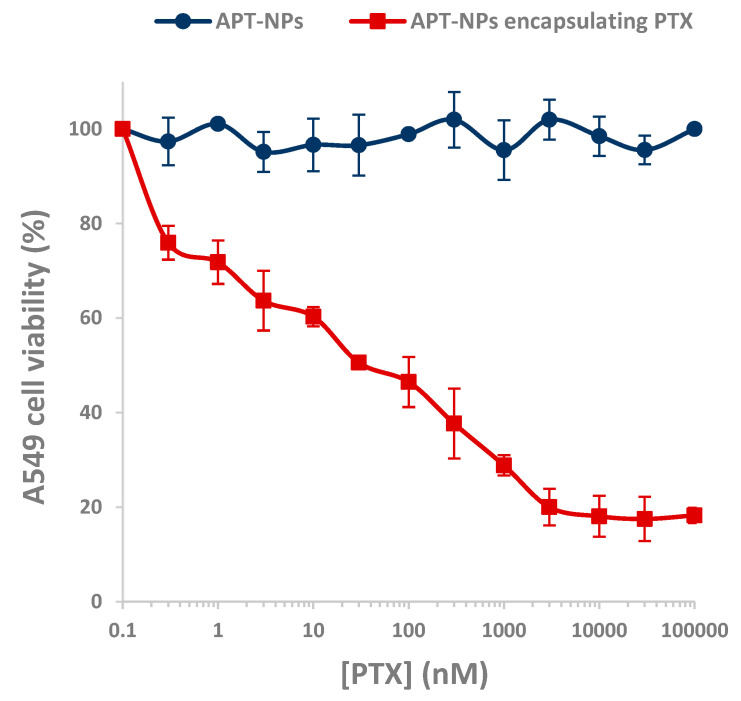
The impact of drug-free APT-NPs vs. APT-NPs encapsulating PTX on the viability of target A549 cells.

Figure 6 shows A549 cells incubated with PTX-lacking or PTX-containing APT-NPs. Drug-free NPs did not show any cytotoxic activity against A549 target cells as opposed to PTX-loaded APT-NPs, which displayed a low IC_50_ value of 30 nM against these target A549 cells. These findings demonstrate that the drug-free APT-decorated NPs are non-toxic.

**Figure 7:**
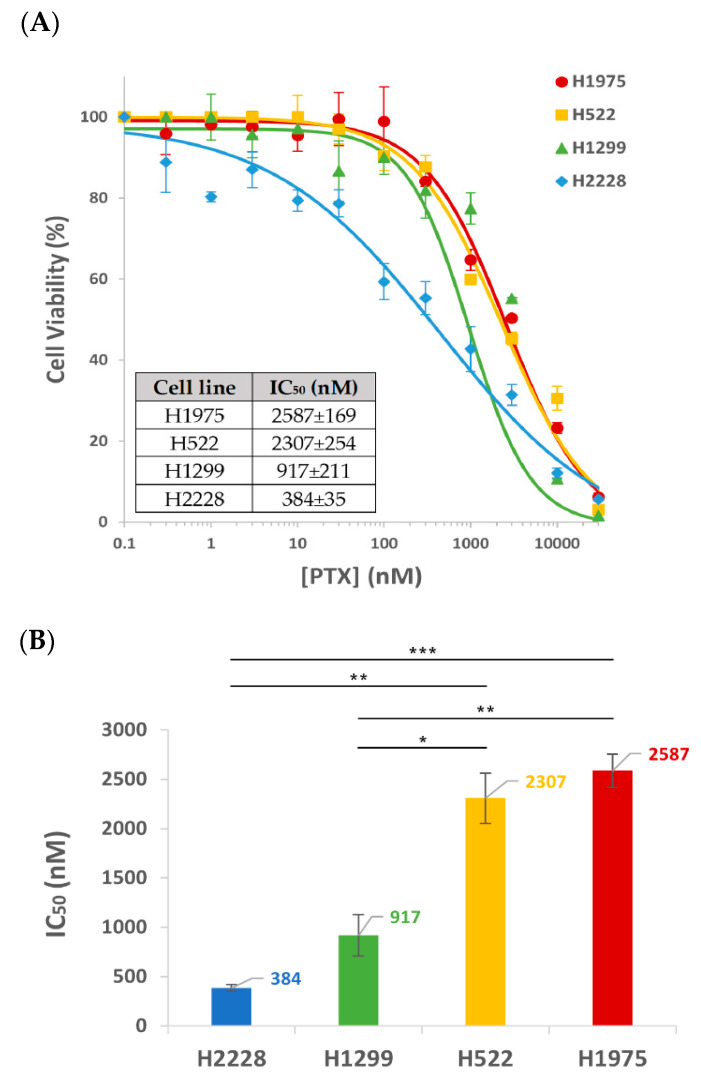
Cytotoxicity of PTX-loaded APT-NPs to various NSCLC cells. (**A**) APT-NPs entrapping PTX were incubated with the NSCLC cell lines H2228, H1299, H522 and H522. Cell viability as a function of the PTX concentration is presented as mean ± SE. (**B**) IC_50_ values derived from each appropriate dose-response curve ± SE. P-values obtained were < 0.05 for all cell lines. * *p* < 0.05, ** *p* < 0.01, *** *p* < 0.001, unpaired, two-tailed *t*-test.

The selectivity and degree of cytotoxic activity of S15-APT-NPs entrapping the chemotherapeutic drug PTX, was determined in the four NSCLC cell lines: H2228, H1299, H1975 and H522. Cell viability was plotted against PTX concentration, accompanied by a fitted dose–response curve (Figure 7A). A similar sigmoidal curve was observed for H1975 and H522 cells, while the curves for H1299 and H2228 were steeper, showing that H2228 displays the steepest cytotoxicity curve. This observation is accompanied by the half maximal inhibitory concentrations (IC_50_) of the encapsulated drug PTX for each NSCLC cell line (Figure 7B). H2228 displayed a remarkable IC_50_ value of 0.38 µM PTX, whereas higher IC_50_ values of 0.92 µM, 2.31 µM and 2.59 µM were obtained with H1299, H522 and H1975 cells, respectively. Although these results reveal some variability in the cytotoxic activity toward the different NSCLC cell lines, these PTX-loaded S15-APT NPs exhibit a relatively potent cytotoxic activity at the sub-micromolar to low micromolar range, toward all the studied NSCLC cell lines.

This difference in the cytotoxic activity between the tumor cell lines also correlated with the internalization findings above, where H2228 cells demonstrated the highest binding and internalization of NPs, hence being the most sensitive to PTX-loaded NPs in this cytotoxicity assay. The H1299 cell line exhibited the second highest IC_50_ value, while showing less binding and internalization ability (Figure 3, Figure 4 and Figure 5). The IC_50_ values of H1975 and H522 were 6–7 fold higher than those of H2228 cells (H1975 cells having the highest IC_50_ value although not statistically different from that of H522). In contrast, a previous study performed in our lab revealed very high IC_50_ values of 87 and 980 μM PTX, entrapped within the same NP delivery system, toward neonatal foreskin fibroblast FSE cells, and human embryonic kidney HEK-293 cells, respectively [38]. These results demonstrate that the PTX-loaded NPs were 34- and 378-fold more cytotoxic toward the least drug-sensitive NSCLC cell line H1975, compared to these healthy cells, respectively.

This comparison of cytotoxicity between the examined NSCLC cell lines, highlights the promising therapeutic potential of the S15-APT NPs drug delivery system (IC_50_ (H1229) = 0.92 μM = 0.79 μg/mL), as it even markedly outperformed an existing EGFR specific small peptide AR-PEG-modified PTX-loaded nanostructured lipid carriers (A-P-NLC) by ~10 fold (IC_50_(H1229) = 7.57 μg/mL), and compared to free PTX reported in that paper, by ~57-fold (IC_50_(H1229) = 45.13 μg/mL) [79]. In this respect, previous studies obtained similar results: the Sgc8c aptamer, which can specifically recognize tyrosine kinase protein 7 (PTK7) on the surface of cancer cells, exhibited high cytotoxicity toward PTK7 receptor-positive CCRF-CEM and HCT116 cell lines, in contrast to receptor-negative K562 and HepG2 cells [80]. Li et al., demonstrated specific recognition of the Sgc8c APT against its target receptor PTK7; therefore, different cytotoxicity levels were observed [80].

The use of a protected S15-APT, with PEG at the 5′ and dT at the 3′ is the same as was applied for Pegaptanib (Macugen), the only aptamer which has been approved by the FDA for clinical use [81]. Pegaptanib is a pegylated anti-vascular endothelial growth factor (VEGF) aptamer for the treatment of age-related macular degeneration (AMD). Therefore, our S15-APT NPs should proceed to in vivo studies, hopefully followed by clinical trials.

Considering the fact that the selective internalization mechanism for target NSCLC A549 cells occurs via classical clathrin-dependent, receptor-meditated endocytosis, as we have previously shown [51], it is herein suggested that the difference observed in the tumor cell lines cytotoxicity was due to different expression levels of the putative S15 APT-specific receptor. The correlation between NPs binding, internalization and cytotoxic activity observed in all NSCLC cell lines in the above experiments suggests that these cell lines express different levels of the putative S15 APT-specific receptor. The higher the levels of this surface receptor expressed by the NSCLC cell line, the higher the binding and the internalization, and consequently the more enhanced the cytotoxicity. These findings imply that the high affinity binding and internalization of the S15-NPs could readily overcome pre-existing mechanisms of anticancer drug resistance such as P-glycoprotein overexpression at the plasma membrane. Thus, these targeted S15-decorated NPs bear important therapeutic implications for the surmounting of cancer MDR by evading plasma membrane-localized MDR efflux pumps that extrude a multitude of structurally and functionally distinct antitumor agents.

These results suggest that the therapeutic efficacy of the developed APT-NPs drug delivery system can be pre-determined depending on the NSCLC tumor that the individual patient is harboring. For such future precision medicine to be applied, further studies should be conducted to identify the APT-specific receptor, which can be used as a strong biomarker for both diagnosis and selectively targeted personalized medicine. The comparison between the different NSCLC cell lines performed in the current research may facilitate finding a correlation between internalization capability of the NPs, to the expression of a specific protein receptor on the plasma membrane of tumor cells, which could allow for a more precisely targeted drug delivery to NSCLC cells and possibly to additional subtypes of LC.

### 3.5. Relation between the Internalization and Cytotoxic Effect of PTX-Loaded NPs on NSCLC Cells

**Figure 8:**
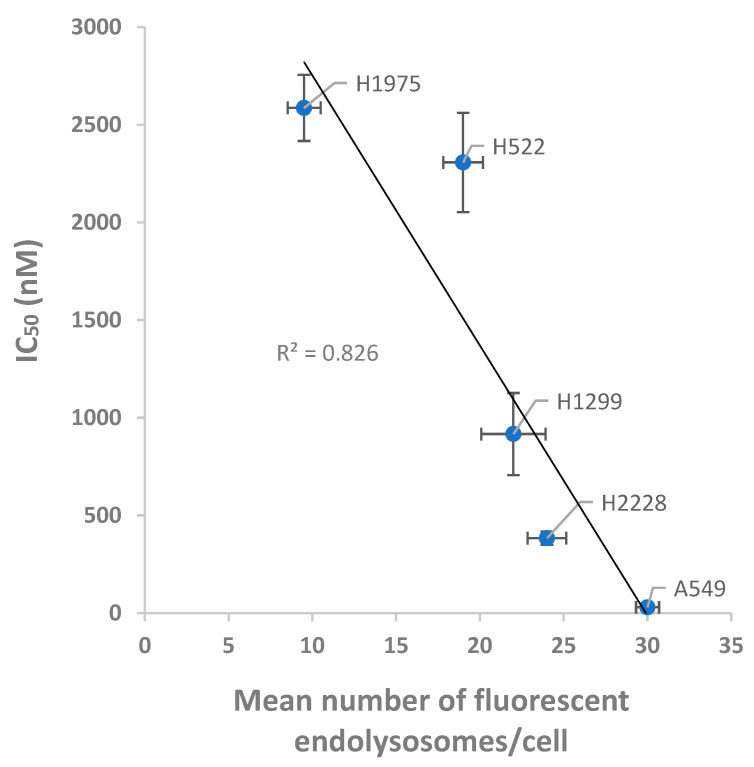
Correlation between the cytotoxic effect to various target NSCLC cells and the internalization ability of PTX-loaded APT-NPs. The IC_50_ value (30 nM) of A549 cells exposed to the same drug delivery system, was determined in our previous study, Engelberg et al. [38].

Results obtained using confocal microscopy and cytotoxicity assays were combined and plotted to explore their correlation (Figure 8). A strong inverse correlation was observed between the number of FEPC and the respective IC_50_ values obtained with the PTX-loaded NPs (R² = 0.826). The higher the number of observed FEPC in the various NSCLC cells, the lower their respective IC_50_ values, which means a better eradication of cancer cells exerted by the PTX-loaded NPs. This observation supports the abovementioned conclusion: the higher the levels of the S15-targeted receptor on the surface of these NSCLC cells, the better is the performance of our S15 APT-NPs drug delivery system in specifically eliminating these target tumor cells.

## 4. Conclusions

To the best of our knowledge, this study is the first to investigate the selectivity and cytotoxicity of APT-decorated NPs to various NSCLC subtypes. Such studies exploring the variability among cancer subtypes are scarce in the literature but are crucial for verifying that targeted NP-based treatments are effective against various subtypes of a given cancer. Our finding of the variable sensitivity of the various NSCLC cell lines to the APT-decorated PTX-containing NPs, supports the hypothesis that the internalization mechanism is based upon a specific interaction of the NPs with a putative plasma membrane receptor expressed on the tumor cell surface. Thus, elevated levels of the receptor in these tumor cells, enhance the specific targeting and cytotoxicity of the APT-NPs. Indeed, the micromolar IC_50_ values we obtained suggest that this receptor is ubiquitously expressed on the surface of lung cancer cells. This implies that our targeted NPs would be efficacious against a large variety of lung cancer types.

## Data Availability

The data presented in this study are available on request from the corresponding authors.

## Supplementary Materials

The following supporting information can be downloaded at: https://www.mdpi.com/article/10.3390/pharmaceutics14081650/s1. Figure S1: Agarose gel electrophoresis of protected vs. unprotected APTs. Figure S2: Selective internalization of protected S15-APTs (with inverted thymidine) into human non-small cell lung A549 target cells. Figure S3: Drug release kinetics.
