# Supplementary material for: Selective Targeting and Eradication of Various Human Non-Small Cell Lung Cancer Cell Lines Using Self-Assembled Aptamer-Decorated Nanoparticles
Source: Pharmaceutics. 2022 Aug 8;14(8):1650. doi: 10.3390/pharmaceutics14081650 (PMC9414336; doi:10.3390/pharmaceutics14081650)
Supplement: Supplementary file 1 [file pharmaceutics-14-01650-s001.zip › pharmaceutics-1803136-supplementary.pdf]

Selective targeting and eradication of various non-small cell lung cancer cell lines using self-assembled aptamer decorated nanoparticles: Supplementary Material

Daniel Barak [1,#], Shira Engelberg [1,#], Yehuda G. Assaraf [2,*] and Yoav D. Livney [1,*]

[1] Lab of Biopolymers for Food & Health, Biotechnology & Food Engineering Dept., Technion, Israel Institute of Technology, Haifa, Israel

[2] Fred Wyszkowski Cancer Research Lab, Biology Dept., Technion, Israel Institute of Technology, Haifa, Israel

[#] Equally contributing first authors

* Correspondence: livney@technion.ac.il; Assaraf@technion.ac.il

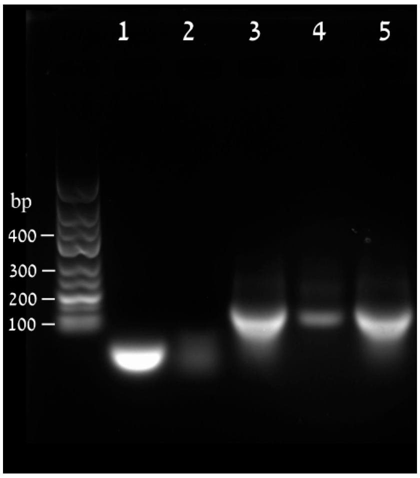

Figure S1:**Agarose gel electrophoresis of protected vs. unprotected APTs**, following incubation with DNase I. Protected and unprotected APTs were incubated for 30 min at 37°C with or without DNase I (10 U/ml). Then, the APTs were resolved by 3% gel electrophoresis in 40 mM Tris-acetate buffer containing 1mM EDTA at pH 8.0. The agarose gel contained the nucleic acid stain GelRed (Biotium). Agarose gel electrophoresis was performed at 100 mV for 30 min and GelRed-stained APTs were visualized under UV light.

The following lanes contained 1μg APT DNA as follows:

1) 25 bps ssDNA control.

2) Non-protected APT after 30 min incubation with 10 U/ml DNase1 at 37°C.

3) Protected APT after 30 min incubation with 10 U/ml DNase1 at 37°C.

4) Non-protected APT.

5) Protected APT.

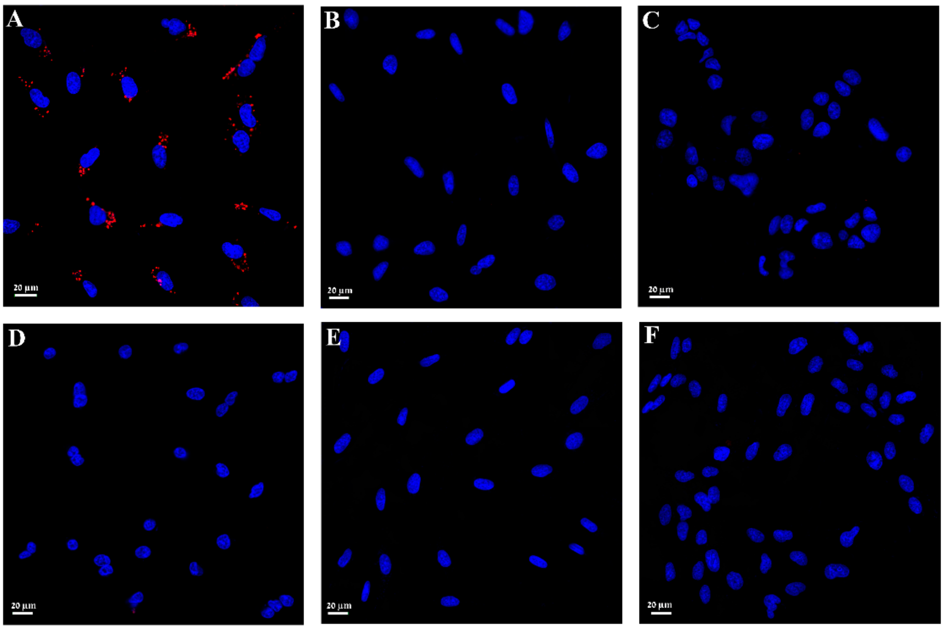

Figure S2: Selective internalization of protected S15-APTs (with inverted thymidine) into human non-small cell lung A549 target cells.

Confocal laser microscopy images of cells exposed to PEG-PCL NPs decorated with 30nM of protected APTs. Samples were diluted 1:5 (v/v) in FBS-free medium and incubated with APT-NPs for 2 h at 37 °C. Protected APTs were labeled with Cy-5 (red). Nuclear DNA was labeled with Hoechst 33342 (2 µg/ ml) (blue).

A) Human non-small cell lung cancer A549 cells.

B) Cervical carcinoma HeLa cells.

C) Colon adenocarcinoma CaCo2 cells.

D) Human embryonic kidney HEK-293 cells.

E) Neonatal foreskin fibroblast FSE cells.

F) Normal human bronchial epithelial BEAS2B cells.

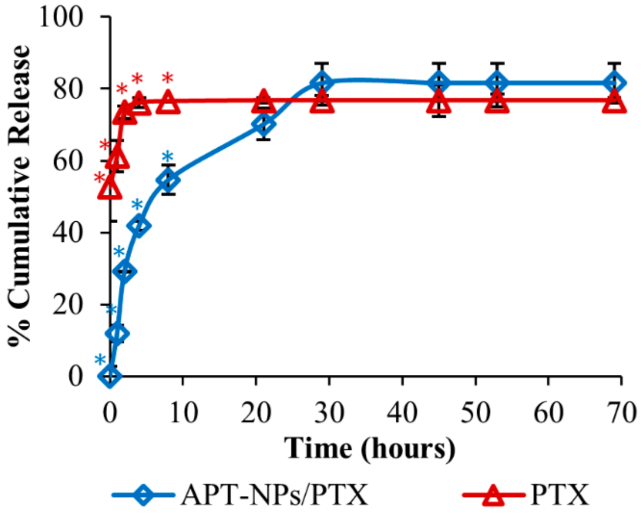

Figure S3:

Drug release kinetics. *In vitro* drug release profile of PTX-loaded APT-NPs (blue line) compared to free drug (red line). One ml of APT-NPs/PTX or free PTX was suspended in 30 mL of PBS at pH 7.4 containing 0.1% Tween 80 and incubated at 37°C with agitation. At designated time points, 30 ml PBS containing Tween 80 were replaced by the same volume of fresh buffer. The samples were freeze-dried and then dissolved in acetonitrile. The amount of PTX at each time point was quantified by HPLC. Data represent means ± SE, n = 3. P-values obtained were <0.001 for 0,1,2,4, and 8 hr of release (marked with *), therefore being significantly different between free PTX and PTX-loaded APT-NPs. These results have been previously published [38].

Reference:

38. Engelberg, S.; Netzer, E.; Assaraf, Y.G.; Livney, Y.D. Selective Eradication of Human Non-Small Cell Lung Cancer Cells Using Aptamer-Decorated Nanoparticles Harboring a Cytotoxic Drug Cargo. Cell Death Dis. 2019, 10, doi:10.1038/s41419-019-1870-0.
